# Acetylome Analysis Identifies SIRT1 Targets in mRNA-Processing and Chromatin-Remodeling in Mouse Liver

**DOI:** 10.1371/journal.pone.0140619

**Published:** 2015-10-15

**Authors:** Sun-Yee Kim, Choon Kiat Sim, Hui Tang, Weiping Han, Kangling Zhang, Feng Xu

**Affiliations:** 1 Singapore Institute for Clinical Sciences, Agency for Science, Technology and Research, Singapore, Singapore; 2 Department of Pharmacology and Toxicology, University of Texas Medical Branch, Galveston, Texas, United States of America; 3 Laboratory of Metabolic Medicine, Singapore Bioimaging Consortium, Agency for Science, Technology and Research, Singapore, Singapore; 4 Department of Biochemistry, Yong Loo Lin School of Medicine, National University of Singapore, Singapore, Singapore; 5 Cardiovascular and Metabolic Disorders Program, Duke-NUS Graduate Medical School, Singapore, Singapore; Northwestern University Feinberg School of Medicine, UNITED STATES

## Abstract

Lysine acetylation is a post-translational modification found on numerous proteins, a strategy used in cell signaling to change protein activity in response to internal or external cues. Sirtuin 1 (SIRT1) is a central lysine deacetylase involved in a variety of cellular processes including metabolism, apoptosis, and DNA repair. Here we characterize the lysine acetylome in mouse liver, and by using a model of *Sirt1* ^*-/-*^knockout mouse, show that SIRT1 regulates the deacetylation of 70 proteins in the liver *in-vivo*. Amongst these SIRT1-regulated proteins, we find that four RNA-processing proteins and a chromatin-remodeling protein can be deacetylated by SIRT1 directly *in-vitro*. The discovery that SIRT1 has a potential role in RNA-processing suggests a new layer of regulation in the variety of functions performed by SIRT1.

## Introduction

Cell signaling is an important aspect of cellular activities in response to internal and external stimuli. Events that involve an increase of protein activity, change in protein subcellular localization, cross-talk with other proteins and pathways often require cell signaling proteins to adopt post-translational modifications of specific amino acid residues [1]. Lysine acetylation is a post-translational modification that was first discovered on histones and later on thousands of non-histone proteins such as transcription factors, binding proteins and enzymes [2]. In metabolic pathways involving glucose and lipid metabolism, the majority of enzymes are acetylated [3]. Acetylation not only changes enzyme activity, but also interactions with other proteins through altered conformation or charge. This effect contributes to energy homeostasis that allows the cell to respond effectively to a changing environment.

Lysine acetylation is a reversible process involving two classes of enzymes that act in opposition to each other: lysine acetyltransferases place an acetyl group on proteins, while lysine deacetylases remove the acetyl group [4]. One class of lysine deacetylases belong to the family of sirtuin deacetylases (SIRT1–7) that are important in stress response, ageing and metabolism [5]. Among the seven sirtuins, we focus on SIRT1 to understand its pathways due to its representative status in the sirtuin family.

SIRT1 is important in a huge diversity of cellular processes such as DNA repair, apoptosis, cell proliferation, metabolism, and cancer [5–7]. A common theme among these functions is that most of SIRT1 functions occur in the nucleus and SIRT1 deacetylates histones or other proteins in the nucleus such as transcription factors or chromatin-remodeling proteins. The deacetylation step requires NAD^+^, a central metabolite that regulates the cellular redox status and is strongly correlated to nutrient availability [8]. SIRT1 has been associated with benefits in metabolism such as improved glucose metabolism, lipid utilization, and insulin sensitivity. Some of these metabolic benefits are, in part, due to direct deacetylation and changes in activity of master transcription factors and coregulators such as PPARα, PGC1α, FOXO1 and SREBP1C [9–13]. Acetylation of proteins related to mRNA transcript splicing can be important in the downstream regulation of transcripts, yet the identity of many of these splicing regulators have not been fully known [14]. Neither is it understood whether these splicing regulators can be specific direct targets of deacetylase SIRT1.

The liver is a major metabolic organ that achieves a huge variety of roles related to detoxication, protein synthesis, and glucose and fatty acid metabolism. For example, SIRT1 is important in glucose metabolism by deacetylating proteins such as PGC1α [12]. The amount of acetylation and deacetyation is highly regulated, as SIRT1 and other sirtuins work closely with acetyltransferases such as TIP60, and in some settings, directly change the acetylation and activity of these acetyltransferases [15]. The deacetylation function of sirtuins can also affect other post-translational modifiers such as methyltransferase SUV39H1 that produce heterochromatin marker lysine 9 methylation on histone H3 to reduce transcription of a large number of targeted genes [16]. Because of the rather complex nature of SIRT1 regulation, we chose to focus on the nucleus to find direct targets of SIRT1 deacetylation. We conducted a proteomic analysis of SIRT1-regulated proteins in the liver and compared the deacetylated proteins in knockout *Sirt1* ^*-/-*^ mice with control *Sirt1* ^*+/+*^ mice. We found a number of RNA-processing co-regulators and chromatin-remodeling factors that are differentially deacetylated and showed that these targets can be specifically deacetylated by SIRT1 *in-vitro*. Our results are similar to a previous acetylome study, suggesting the validity of our data [17]. Furthermore, we have found additional SIRT1-regulated proteins that could be important in the myriad of functions achieved by SIRT1 in the nucleus. These SIRT1 substrates could be further tested in conjunction with known key regulators in the liver, especially by using point mutations that disrupt the putative deacetylated lysine residues.

## Materials and Methods

### Animal Study and Ethics Statement

*Sirt1* knockout mice (*Sirt1* ^-/-^) were created by crossing *Sirt1*-floxed mice [18] to mice expressing a ubiquitously expressed mER-Cre (GSK-licensed) to delete 51 amino acids at exon 4 of *Sirt1* gene. The resulting truncated SIRT1 protein is catalytically inactive [18]. *Sirt1* control mice (SIRT1^+/+^) were *Sirt1*-floxed mice without the Cre driver. All mice were in C57BL/6 background and housed at room temperature (25°C). Both the SIRT1^+/+^ and *Sirt1* ^*-/-*^ mice were maintained under the same conditions in this study. Before tissue harvesting, all mice were fasted for 24 hours to enhance SIRT1 activity. All animals were maintained on a normal chow diet at Sirtris, a GSK Company (Cambridge, MA) following the guidelines of the institutional animal use and care committee (IACUC). All protocols and animal ethics were approved by the Sirtris IACUC. The animals were sacrificed by asphyxiation using carbon dioxide followed by cardiac puncture.

### Preparing Liver Extract and In-Solution Tryptic Digestion

To prepare proteins for mass spectrometry analysis, frozen liver tissues of three *Sirt1* ^*+/+*^ control mice and *Sirt1* ^*-/-*^ mice were homogenized in PLC lysis buffer (50 mM HEPES [pH = 7.5], 150 mM NaCl, 10% glycerol, 1 mM EGTA, 1% triton x-100) containing trichostatin A and protease inhibitor cocktail (Roche). Six milligrams of liver extracts were resuspended in 50 mM NH_4_HCO_3_ [pH 8.5] using buffer exchange column (Bio-spin column, Biorad) and trypsinized with sequencing-grade trypsin (V5111, Promega) at an enzyme-to-substrate ratio of 1:50 for 16 hours at 37°C. Digested peptides were reduced with 5 mM DTT at 50°C for 30 min and alkylated with 15 mM iodoacetamide at room temperature for 30 min in darkness, and then quenched by 15 mM cysteine at room temperature for 30 min. Additional trypsin was added at an enzyme-to-substrate ratio of 1:100 for 4 hours at 37°C to ensure complete digestion. Trypsinized peptides were dried in a speedvac for 30 min at 30°C. To prepare acK-peptide standard, acetylated bovine serum albumin (BSA) (ICP6090, Immunechem) was trypsinized using the same method.

### Immunoprecipitation with Pan-acetyl-lysine Antibody

The digested peptides were resolubilized in NETN buffer (50 mM Tris-HCl [pH = 8.0], 1 mM EDTA, 100 mM NaCl, 0.5% NP-40, and protease inhibitor cocktail (Roche)) and incubated with agarose bead coupled to anti-acetyl-lysine antibody (ICP0388, Immunechem) for overnight at 4°C with shaking. The acetylated peptides bounded to the beads were washed three times with NETN buffer and twice with ENT buffer (50 mM Tris-HCl [pH = 8.0], 1 mM EDTA, 100 mM NaCl), then eluted from the beads by washing three times with 1% trifluoroacetic acid then dried completely. For normalization, 100 fmol of trypnisized acetylated BSA peptides were added.

### LC-MS/MS analysis of Immunoprecipitated Peptides

The pan-acetyl-lysine antibody pull-down samples were desalted with a hypercarb Toptip column (Poly LC, MD, USA). The eluted peptides with 60% acetonitrile (ACN) were vacuum-dried, reconstituted in 30 μl of 0.1% FA, and then subjected to LC-MS/MS analysis. Peptides were separated by online reverse phase liquid chromatography (RPLC) using an Easy-nLC equipped with an autosampler (Thermo Scientific). A Thermo Acclaim PepMap RSLC, 50 μm x 15 cm, 2-μm nanoViper C18, 100 Å analytical column was used for the separation. A precolumn (Thermo, 75 μm × 2 cm, 3 μm C18, 100 Å) was brought in line with the analytical column and a 120-min gradient (solvent A, 0.1% FA in water; solvent B, 0.1% FA in ACN) from 5–30% solvent B was used for separating the peptides. The Q-Exactive mass analyzer was set to acquire data at 35,000 FWHM resolution for parent full-scans followed by data-dependent high collision-energy dissociation (HCD) MS/MS of the top 12 most abundant ions acquired at 17500 FWHM.

### Peptide Identification from LC-MS/MS data

Proteins were identified and quantified through the Proteome Discoverer 1.4 platform (thermo) by using Sequest HT searching engine and the *mouse* database. Sequest parameters were used as follow: Carbamidomethylation of cysteine and acetylation of lysine were set as fixed modifications and oxidation of methionine and deamination of asparagine (D) and glutamine (Q) were set as variable modifications. Trypsin was the protease selected and up to two missed cleavages was used. Mass tolerance for the precursor ions was 10 ppm and for the MS/MS 0.2 Da. Only peptides with minimal length of four amino acids were considered and peptides were filtered for maximum false discovery rate of 1%. Acetylated peptides were further manually confirmed by comparing their detected fragmentation ions with theoretic counterparts and guaranteeing the appearance of *m/z* 126.0914 corresponding to acetylated lysine.

### Immunoblotting and Immunoprecipitation

Liver tissues were homogenized with PLC lysis buffer containing protease inhibitor cocktail and cleared by centrifugation at 12,000 rpm for 15 min. Protein concentration was determined using BCA method (Sigma). Equal amount of lysate from mouse liver extracts were resolved by SDS-PAGE, transferred onto PVDF membranes, and probed with antibodies recognizing the carboxy terminus of SIRT1 (#2028, Cell Signaling), Tubulin (#05–661, Millipore), Histone H2A (#2595, Cell Signaling), hnRNP L (ab6106, Abcam), and hnRNP C1/C2 (ab10294, Abcam). For immunoprecipitation (IP) assay, 1 mg of tissue extracts was incubated with pan-acetyl-lysine antibody immobilized to agarose beads (ICP0388, ImmuneChem) for overnight at 4 degree followed by washing with lysis buffer for six times. The resulting immunoprecipitants were eluted using 2X laemmli sample buffer and subjected to western blotting analysis using the indicated antibodies.

### *In-vitro* Deacetylation Assay

Deacetylation assay was performed using recombinant SIRT1 (#524743, Calbiochem) and a SIRTainty Class III HDAC Assay Kit (Millipore) on synthetic acetyl-lysine peptide substrates (Mimotopes) of heterogeneous nuclear ribonucleoprotein L acetylated at K62, Isoform 1 of Zinc finger protein 638 acetylated at K1302, Isoform 2 of RNA-binding protein 10 acetylated at K54, Isoform 3 of heterogeneous nuclear ribonucleoproteins C1/C2 acetylated at K240, SWI/SNF complex subunit SMARCC2 isoform 2 acetylated at K769, U4/U6.U5 tri-snRNP-associated protein 1 acetylated at K11. The levels of lysine deacetylation were determined by measuring the intensities of the fluorescent signals generated by free ammonia using a fluorescence plate reader (Tecan).

### Protein Ontology analysis

Protein ontology was analyzed using Uniprot/Swiss-Prot and Database for Annotation, Visualization, and Integrated Discovery (DAVID).

### Statistics

Results are expressed as mean ± SEM. The difference between two mean values was compared using two-tailed Student’s *t*-test. A *P* value of < 0.05 was considered statistically significant. All experiments were performed at least three times.

## Results

### Experimental strategy for identifying the lysine acetylome in *Sirt1* ^*+/+*^ and *Sirt1* ^*-/-*^ mice

**Fig 1:**
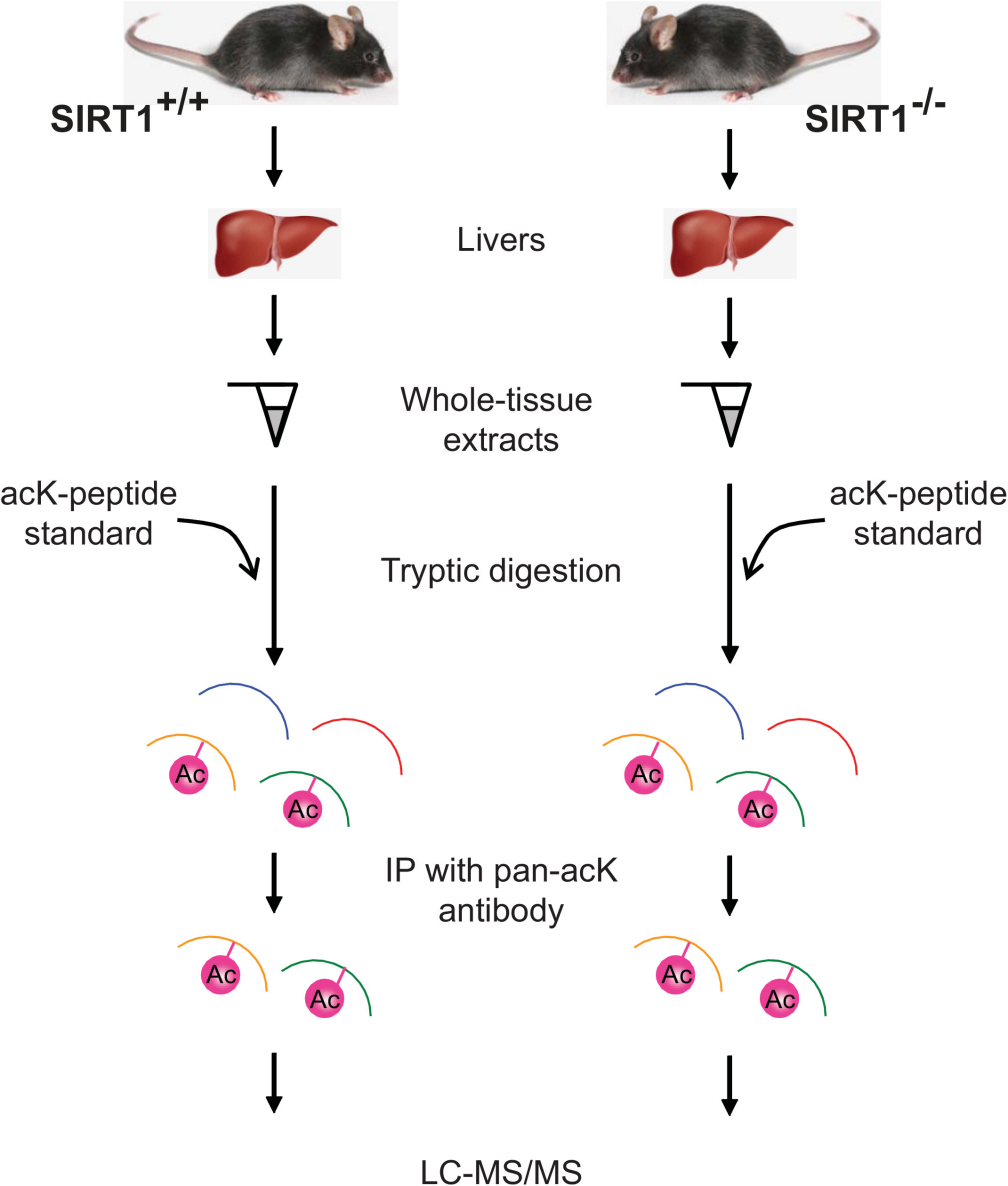
Strategy for identifying SIRT1-regulated acetylome in mouse liver. Mouse livers were isolated from control SIRT^+/+^ and knockout SIRT^-/-^ male mice. Proteins were digested with trypsin and the resulting peptides were immunoprecipitated using a pan-acetyl-lysine antibody. An acetylated lysine (acK) standard was “spiked in” as an internal control. Enriched acetyl lysine peptides were analyzed by LC-MS/MS.

Lysine acetylome has been previously characterized in liver tissues [3, 17, 19], but how much of this acetylome is regulated by SIRT1 remains unknown (also see [20]). Here we focused on a subset of the liver acetylome that is regulated by SIRT1 by comparing the acetylome between *Sirt1* ^*-/-*^ and control (*Sirt1* ^*+/+*^) mice (Fig 1). Liver tissues dissected from *Sirt1* ^*+/+*^ and *Sirt1* ^*-/-*^ mice were used for proteomic analysis using mass spectrometry to find a comprehensive collection of acetylated proteins. Briefly, the tissues were digested with trypsin that cleaves peptides at lysines or arginines only when they are not acetylated (Fig 1). Hence the ends of the resulting peptides are not acetylated lysines but any lysines found in the middle of the cleaved peptides are acetylated and are what we analyzed further. As an internal control, a fixed amount of acetylated bovine serum albumin (BSA) peptides were added. To enrich the peptides containing acetyl lysines, peptides treated with trypsin were immunoprecipitated using an anti- acetyl-lysine antibody and analyzed by liquid chromatography-tandem mass spectrometry (LC-MS/MS). The peptides found by LC-MS/MS were identified by comparing to the mouse database using SequestHT.

### Identification of Acetylated Proteins in *Sirt1* ^*+/+*^ and *Sirt1* ^*-/-*^ Mouse Livers

**Fig 2:**
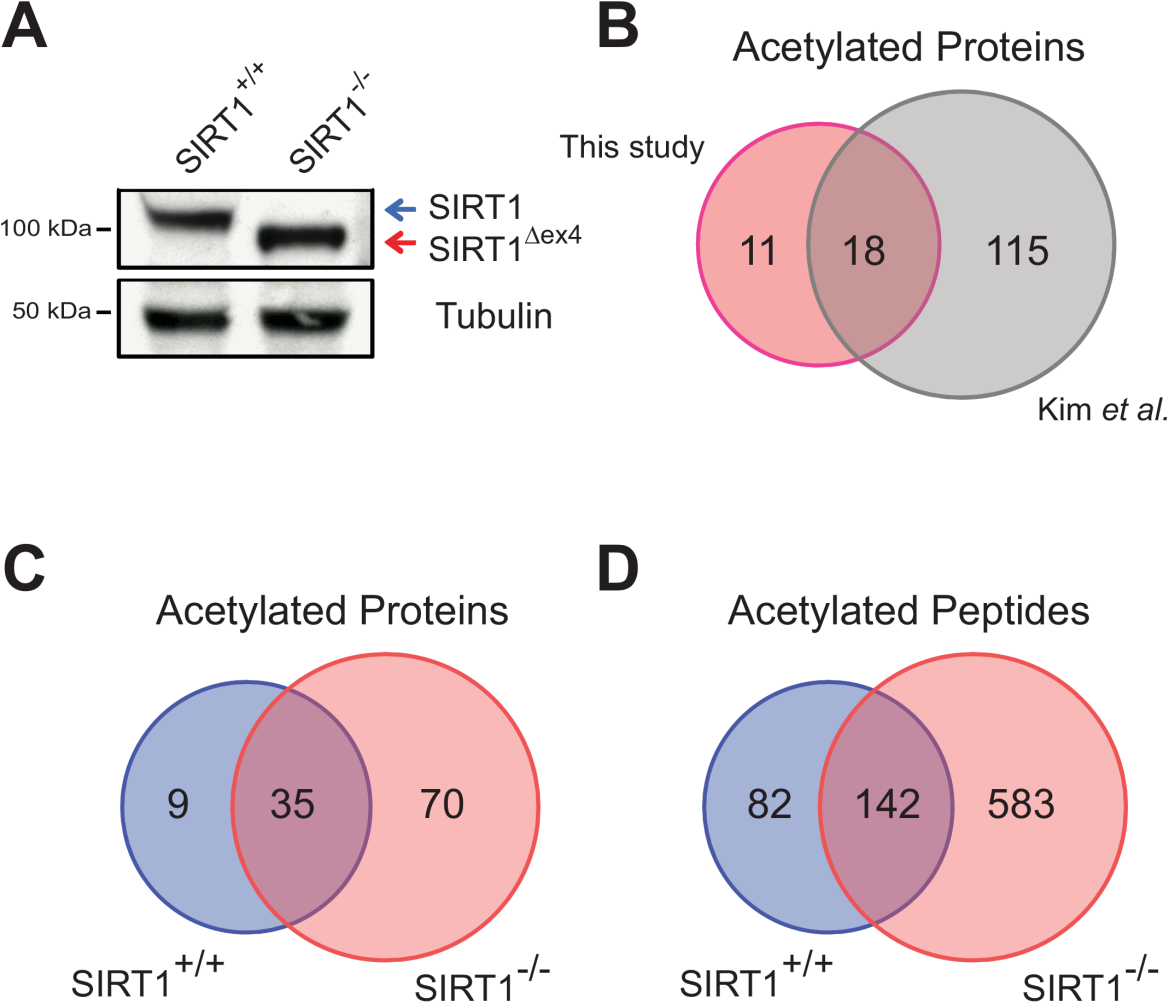
Identification of acetylated proteins and peptides by LC-MS/MS. **(A)** Western blot analysis of SIRT1 and loading control Tubulin in liver extracts from SIRT1^+/+^ and SIRT1^-/-^ mice. Blue arrow depicts the full-length SIRT1 protein while red arrow refers to the truncated mutant SIRT1 protein. **(B)** Venn diagram comparing acetylome studies: this study and Kim *et al*. [17]. **(C** and **D)** Venn-diagrams showing the acetylated proteins and peptides from SIRT^+/+^ and SIRT1^-/-^ mice in our study.

To verify that the *Sirt1* mutation induced by the Cre driver is complete in the liver, western blot analysis was conducted using an antibody against SIRT1 using the liver samples from control or *Sirt1* ^-/-^ mice. We found that the normal-sized SIRT1 protein was completely absent in the knockout *Sirt1* ^*-/-*^ sample and instead there was a smaller SIRT1 protein, suggesting that exon 4 of *Sirt1* gene had been efficiently deleted (Fig 2A). This smaller SIRT1 protein has defective deacetylation activity as reported previously [18].

To test whether our acetylome results are reliable, we compared our data to a previously published acetylome study [17]. Kim *et*.*al*. used the mitochondrial fraction of wild-type mouse livers to perform a lysine acetylome study. For fair comparison, we looked at a subset of our control mice data and filtered for only the mitochondrial proteins. Comparison of the two datasets revealed that 18 of 29 proteins (or 62%) in our study are also found in the Kim *et*.*al*. data (Fig 2B). The close similarity of the data suggests that our acetylome results are credible.

To find SIRT1-regulated proteins, we analyzed our data for peptides that were enriched in acetyl lysines in *Sirt1* ^*-/-*^ but not in control mice. As expected from the inhibition of a protein deacetylase, mice deficient for SIRT1 activity show hyperacetylation of a broad range of proteins. Out of a total of 807 acetyl lysine peptides from 114 proteins identified by mass spectrometry (S1 Table) in the two genotypes, a large majority comprising 583 peptides from 70 proteins is only found in *Sirt1* ^*-/-*^ but not in control mice (Fig 2C and 2D).

**Fig 3:**
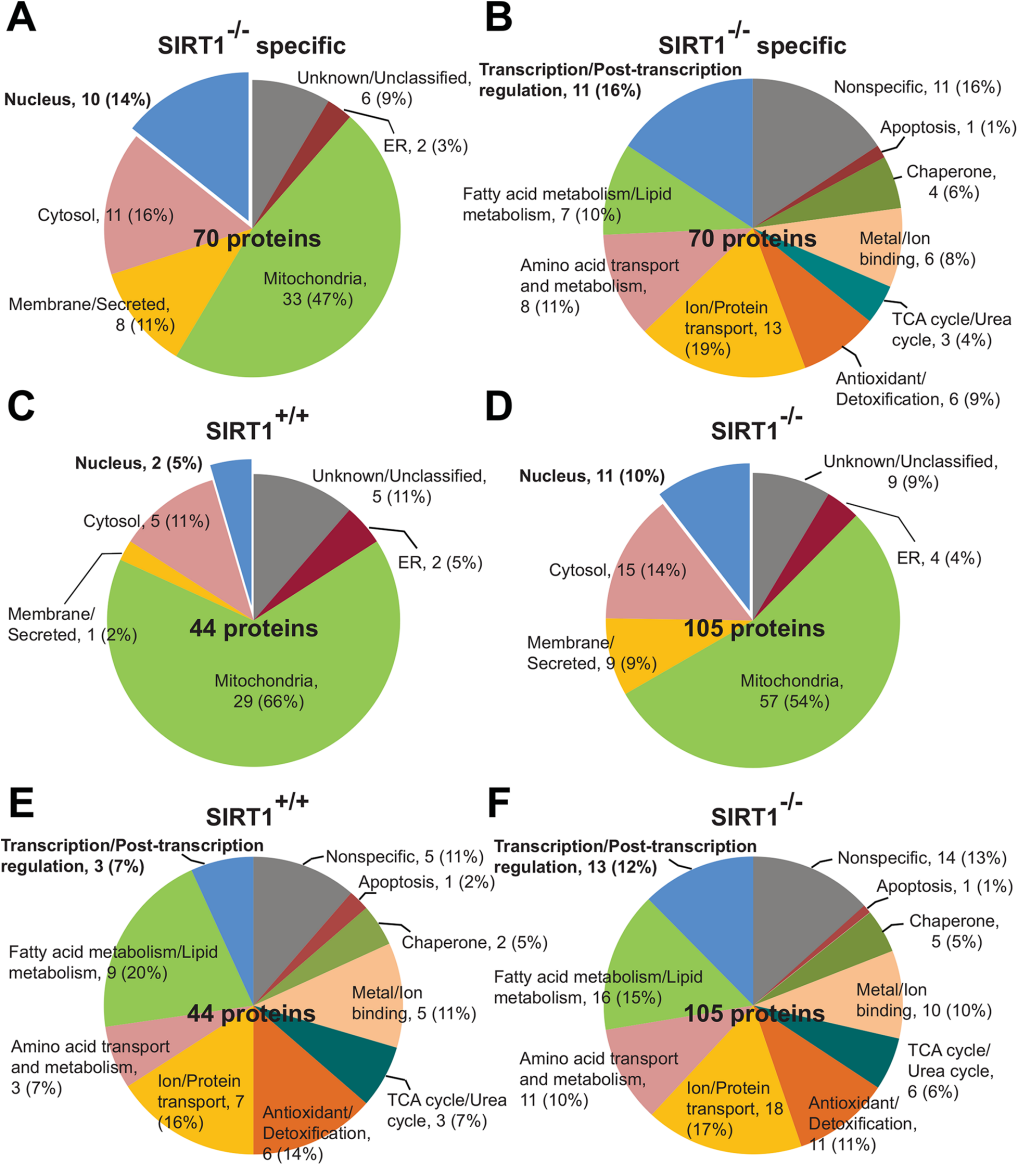
Protein ontology of acetylated proteins. Subcellular localization **(A)** and biological process **(B)** of 70 SIRT1^-/-^ specific proteins that are not shared with SIRT1^+/+^ (also refer to Fig 2C). Subcellular localizations **(C** and **D)** and biological processes **(E** and **F)** were also shown for SIRT1^+/+^ and SIRT1^-/-^ mice.

To better understand the biological function of these 70 differentially regulated proteins, protein ontology was used for classification by subcellular localizations and biological processes (Fig 3A and 3B). The proteins were categorized into: transcription/post-transcription regulation (16%), fatty acid metabolism (10%), amino acid metabolism (11%), ion and protein transport (19%), antioxidant and detoxification (9%), TCA/urea cycles (4%), metal and ion binding (8%), chaperone (6%), and apoptosis (1%) (Fig 3B). Most of the proteins (10 out of 11 proteins) with roles in transcription / post-transcription regulation are found in the nucleus and represent our main focus as SIRT1 is predominantly nuclear. In addition, cytosolic target proteins are also present and we think that these are downstream indirect targets of SIRT1. These cytosolic proteins are divided into categories of antioxidant and detoxification, metal and ion binding, and amino acid metabolism. Interestingly, mitochondrial proteins represented the biggest cluster (47%) of SIRT1-regulated proteins, many of which are proteins involved in tricarboxylic acid (TCA) cycle, fatty acid oxidation, ion/electron transport (Fig 3A and 3B). This is perhaps a little surprising as *SIRT1* protein is commonly known to exert its function in the nucleus. However, we note that it has recently been shown that SIRT1 also affects the function of a mitochondrial deacetylase SIRT3 [21], suggesting that some of the mitochondrial proteins that we have identified to have altered acetylation patterns in *Sirt1* ^*-/-*^ might have been mediated through SIRT3.

Because the differentially regulated proteins are found in all regions of the cell (Fig 3A), we asked whether it is appropriate to extend our further analyses to regions other than the nucleus. We took a broader view of the total proteins identified in *Sirt1* ^*-/-*^ and control mice and found that the removal of SIRT1 activity appeared to have little change in the proportion of most classes of subcellular localizations and biological processes, suggesting that the pleiotropic effects are most likely indirectly regulated by SIRT1 (Fig 3C to 3F). The main exception where the proportional change is the biggest is in the nucleus and in transcription/post-transcription function, which is consistent with current paradigm that SIRT1 has a direct nuclear role. Hence, we restricted our further analyses to SIRT1-regulated nuclear proteins.

### Identification of New SIRT1 Deacetylation Targets

**Fig 4:**
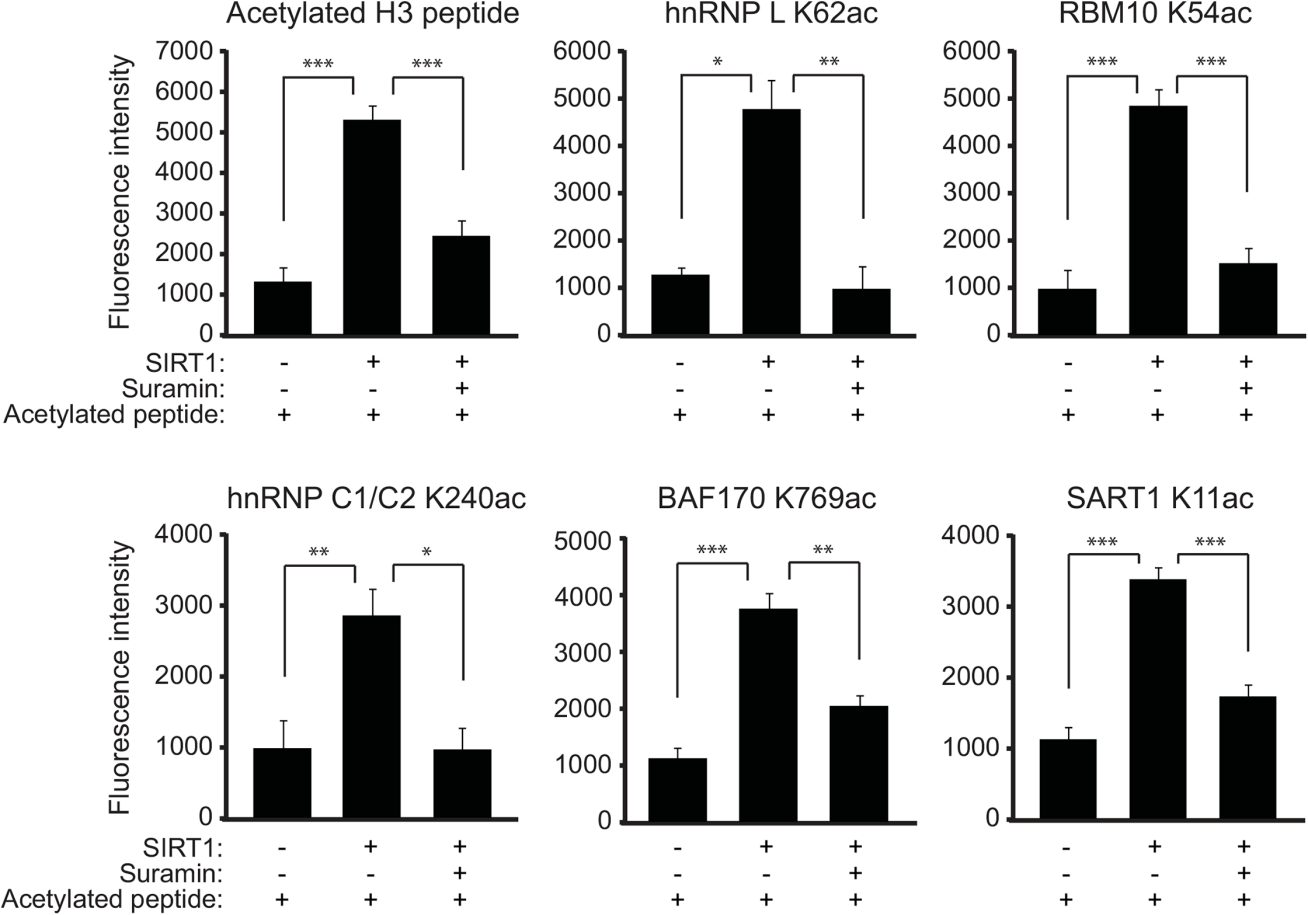
Validation of SIRT1 target peptides using *in-vitro* deacetylation assay. Peptides examined in this assay were: (1) Acetylated H3 peptide (positive control), (2) Heterogeneous nuclear ribonucleoprotein L (hnRNP L) K62ac, (3) Isoform 2 of RNA-binding protein 10 (RBM10) K54ac, (4) Isoform 3 of Heterogeneous nuclear ribonucleoproteins C1/C2 (hnRNP C1/C2) K240ac, (5) SWI/SNF complex subunit SMARCC2 isoform 2 (BAF170) K769ac, (6) U4/U6.U5 tri-snRNP-associated protein 1 (SART1) K11ac. Suramin is a broad-spectrum sirtuin inhibitor. Fluorescence level measured the amount of deacetylation. Graphs in this figure are representative of three independent experiments (mean ± SEM) (*P <0.1, **P <0.01, and ***P < 0.001).

**Fig 5:**
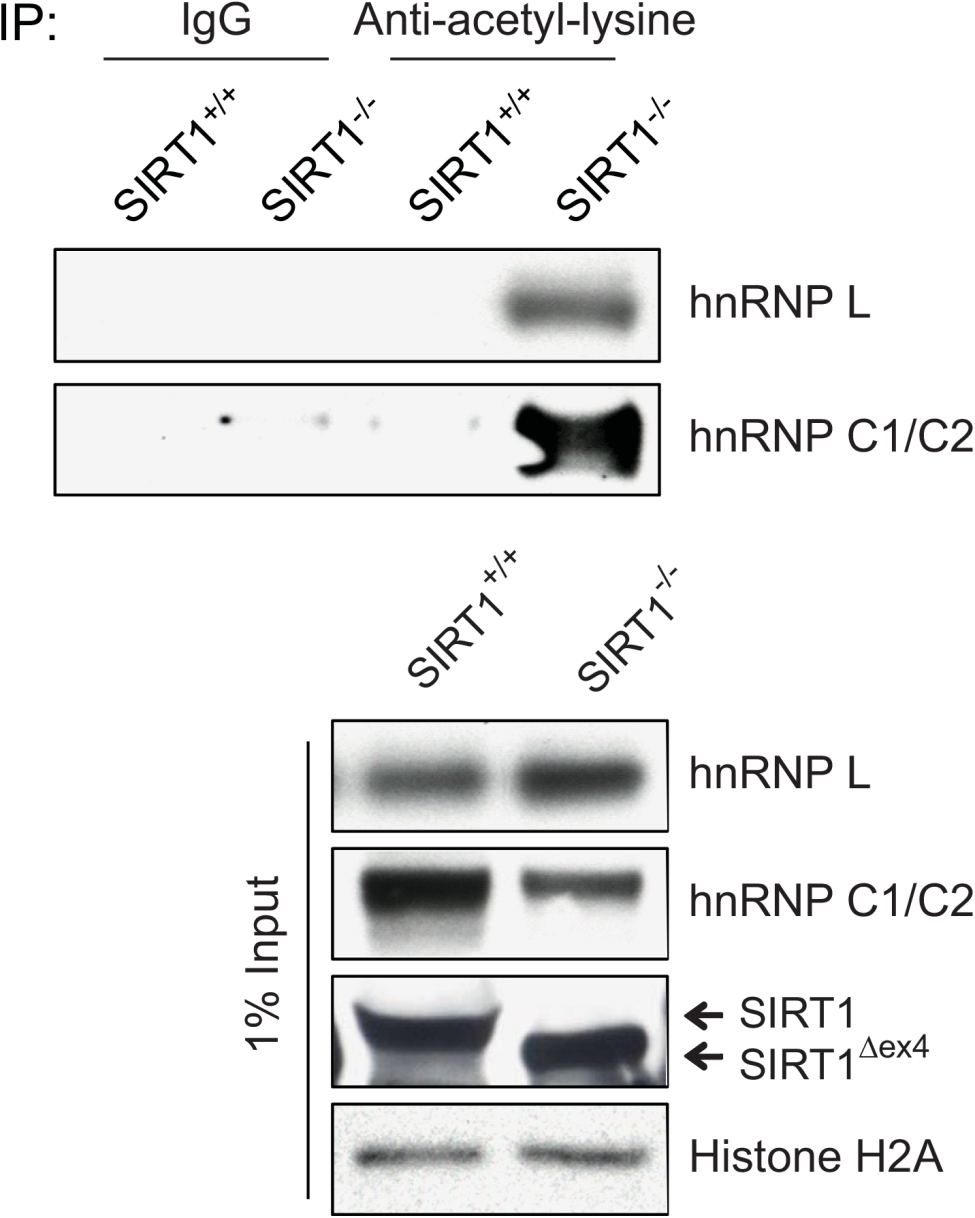
SIRT1 deacetylates hnRNP L and hnRNP C1/C2 *in vivo*. Acetylated proteins were immunoprecipitated using the pan-acetyl-lysine antibody from the liver extracts of SIRT1^+/+^ and *Sirt1* ^*-/-*^ mice. The amounts of acetylated hnRNP L and hnRNP C1/C2 proteins were evaluated by their corresponding antibodies in the IP samples. The protein levels of hnRNP L, hnRNP C1/C2 and SIRT1 were assessed by western blotting using the corresponding antibodies. Histone H2A was used as a loading control.

Given the discovery of acetyl lysine peptides in the liver extracts from *Sirt1* ^*-/-*^ mice, we next questioned whether the candidate peptides could be deacetylated by SIRT1 directly *in-vitro*. There were in total ten nuclear proteins comprising 38 acetylated peptides (Fig 3A and Table 1). All except four peptides contained acetyl lysine sites that were novel and not found in existing databases Uniprot and PhosphoSitePlus (www.phosphosite.org). Of the 38 acetylated nuclear peptides, we selected six peptides that were abundant in the LC-MS/MS data (Table 2). Synthetic acetyl lysine peptides were generated for these six candidates and an *in-vitro* deacetylation assay was used to test whether the acetylation modifications can be removed by SIRT1. Out of the six candidate peptides, we found that in the presence of NAD^+^, a group of five that included heterogeneous nuclear ribonucleoproteins hnRNP L K62ac and hnRNP C1/C2 K240ac, U4/U6.U5 tri-snRNP-associated protein SART1 K11ac, RNA-binding proteinRBM10 K54ac, and SWI/SNF complex subunit SMARCC2/BAF170 K769ac that showed significant deacetylation by SIRT1 (Fig 4). This deacetylation was rescued by suramin, a broad-spectrum sirtuin inhibitor, suggesting that the deacetylation is specific to sirtuins [22]. These proteins are associated with RNA-processing or chromatin-remodeling, and particularly for RNA-processing, suggested a new layer of regulation by SIRT1. Positive control acetylated histone H3 peptide showed a similar acetylation pattern as the other five peptides, suggesting that the assay was robust (Fig 4). The sixth peptide ZNF638 K1302ac could not be deacetylated by SIRT1 (data not shown). To further verify these observations *in vivo*, we used the liver extracts prepared from SIRT1 WT and *Sirt1* ^*-/-*^ mice. We immunoprecipitated the acetylated proteins using the pan-acetyl-lysine antibody and then we evaluated the amount of specific acetylated proteins in the IP samples by their corresponding antibodies. As shown in Fig 5, we observed significantly more acetylated hnRNP L and hnRNP C1/C2 proteins in *Sirt1* ^*-/-*^ mice as compared with the WT mice. These results suggest that regulation by SIRT1 in the nucleus can extend to RNA-processing and raise the possibility that such regulation can be crucial to the metabolic benefits that SIRT1 provides in the liver.

**Table 1:** List of the acetylated nuclear proteins identified only in SIRT1^-/-^ mice liver.

Accession number	Protein name	Peptide sequence	Acetylation site	Novel site?
IPI00620362.4	Heterogeneous nuclear ribonucleoprotein L	L**k**TENAGDQHGGGGGGGSGAA GGGGGENYDDPHK	K62	Yes
		**k**NGVqAmVEFDSVQSAQRAK	K226	Yes
IPI00222208.2	Heterogeneous nuclear ribonucleoprotein U-like protein 2	AVEEQGDDQDSE**k**SK	K185	Yes
		**k**EVEGDDVPESImLEmK	K575	Yes
		L**k**VTELRSELQR	K7	No
IPI00330763.3	Isoform 1 of Protein FAM76B	SSAAIqnETPK**k**K	K218	Yes
		**k**LTEL**k**ADFQYqESNLR	K276, K281	Yes
		T**k**MnSmE**k**AHK	K294, K300	Yes
		**k**YGAPqTcEqcK	K96	Yes
IPI00134457.3	Isoform 1 of Protein SON	LTDLD**k**AqLLEIAK	K2073	No
		**k**N**k**NR	K1830,K1832	Yes
IPI00608012.1	Isoform 1 of Zinc finger protein 638	DIAE**k**VILDEK	K1302	Yes
		**k**PqNK	K753	Yes
		SVVTVAA**k**G**k**ASIK	K825, K827	Yes
		**k**EnETPR	K477	Yes
		DDSNN**k**ALALQNTK	K1505	Yes
		**k**PSDIkkSSASALK	K762	Yes
IPI00468834.4	Isoform 2 of RNA-binding protein 10	EYGSQEG**k**HEYDDSSEEQSAEIR	K54	Yes
		**k**QGIVTPIEAqTR	K881	Yes
		GS**k**RDMASnEGSR	K386	Yes
		E**k**cFK	K232	Yes
IPI00223444.1	Isoform 3 of Heterogeneous nuclear ribonucleoproteins C1/C2	NE**k**SEEEQSSASVK	K240	Yes
IPI00453826.2	Matrin-3	TDAQ**k**TESPAEGK	K616	No
		HmQ**k**GR	K391	No
IPI00651846.1	SWI/SNF complex subunit SMARCC2 isoform 2	PEGQAADE**k**K	K769	Yes
		ESE**k**SDGDPIVDPE**k**DK	K812, K823	Yes
		E**k**PADmqnFGLR	K568	Yes
		**k**GGnYK	K292	Yes
		**k**ISA**k**TLTDEVnSPDSDR	K271, K275	Yes
		S**k**RGHR	K328	Yes
		**k**KISAK	K270	Yes
IPI00323674.6	U4/U6.U5 tri-snRNP-associated protein 1	GE**k**EAAGTTAAAGTGGTTEQPPR	K11	Yes

**Table 2:** List of the acetylation sites tested in the SIRT1 *in vitro* deacetylation assay (nuclear proteins).

Accession #	Uniprot #	Protein name	Peptide sequence	Acetylation site	Cellular component	Biological process
IPI00620362.4	Q8R081	Heterogeneous nuclear ribonucleoprotein L (hnRNP L)	APKRL(**AcK**)TENAG	K62	Cytoplasm, Nucleus	mRNA processing
IPI00608012.1	Q61464	Isoform 1 of Zinc finger protein 638 (ZNF638)	KDIAE(**AcK**)VILDE	K1302	Nucleus	Transcription, Transcription regulation
IPI00468834.3	Q99KG3	Isoform 2 of RNA-binding protein 10 (RBM10)	GSQEG(**AcK**)HEYDD	K54	Nucleus	3'-UTR-mediated mRNA stabilization
IPI00223444.1	Q9Z204	Isoform 3 of Heterogeneous nuclear ribonucleoproteins (hnRNP C1/C2)	EMKNE(**AcK**)SEEEQ	K240	Nucleus, Spliceosome	mRNA processing, mRNA splicing
IPI00651846.1	Q6PDG5	SWI/SNF complex subunit SMARCC2 isoform 2 (BAF170)	QAADE(**AcK**)KEPKE	K769	Nucleus	Transcription, Transcription regulation
IPI00323674.6	Q9Z315	U4/U6.U5 tri-snRNP-associated protein 1 (SART1)	KHRGE(**AcK**)EAAGT	K11	Nucleus, Spliceosome	mRNA processing, mRNA splicing

Although the majority of the previous studies were focused on the role of SIRT1 in regulating nuclear proteins, there were several reports on SIRT1’s function in cytoplasm and mitochondria [23–27]. Therefore, we have selected seven candidate sites (S2 Table) for SIRT1 deacetylation from seven non-nuclear proteins in our list and examined the SIRT1 deacetylase activity on these substrates *in vitro*. As shown in S1 Fig, SIRT1 deacetylated HADH K202ac and CPT2 K93ac in the *in vitro* assay but not the other five candidate sites. Whether these two acetylation sites were direct targets of SIRT1 deacetylation *in vivo* remained to be determined.

## Discussion

The liver is a key metabolic organ regulating lipid and glucose metabolism in mammals. In response to nutritional and hormonal signals, the liver switches between modes of energy storage and energy production to maintain metabolic homeostasis in humans. SIRT1 plays a central role in the regulation of hepatic glucose and lipid metabolism by deacetylating various targets whereby modulating their molecular functions. For example, during short-term fasting, SIRT1 increases and leads to reduced TORC2 acetylation and decreased gluconeogensis [28]; during prolonged fasting, SIRT1 deacetylates and activates PGC-1α to promote fatty acid oxidation and glucose homeostasis [9, 12, 29, 30]. In addition, SIRT1 promotes gluconeogenesis by deacetylating and activating FoxO1 in liver [31]. Through deacetylation of the SREBP proteins, SIRT1 also regulates hepatic lipid metabolism [11, 32].

To explore further and systematically for novel SIRT1 targets in the liver, we conducted a comprehensive study to identify the lysine acetylome of knockout *Sirt1* ^*-/-*^ and control *Sirt1* ^*+/+*^ mouse livers. Our results are consistent with a previous acetylome study [17], as the two datasets shared a large overlap of 62% of the proteins found in our study. Moreover by comparing the results from *Sirt1* ^*+/+*^ to a model of *Sirt1* ^*-/-*^ mouse, we showed the SIRT1 is important for the deacetylation of 70 proteins of wide-ranging functions. We further pursued the putative SIRT1 direct targets in the nucleus by testing them using *in-vitro* deacetylation assays to show that the candidates can be deacetylated by SIRT1 protein. We verified that four RNA-processing proteins and a chromatin-remodeling protein are new SIRT1 deacetylation substrates.

The discovery that SIRT1 can deacetylate RNA-processing proteins is particularly interesting. hnRNP C1/C2 and hnRNP L belong to the family of ~20 hnRNP proteins that are highly abundant in the nucleus and play a crucial post-transcriptional role in RNA splicing, nuclear-cytosolic shuttling and translation [33]. Subunits of hnRNP complex are added or removed in a dynamic fashion as the RNA transcript is spliced, exported to the cytoplasm and associate with the translation machinery. These proteins are found in most tissues, but at a different level. hnRNP proteins are known to have post-translational modifications such as phosphorylation, sumoylation, ubiquitination, and methylation that can affect the protein stability and cellular localization [34]. Acetylated hnRNP A1 protein has been previously found [17] and a recent paper also showed the importance of acetylation in affecting the splicing activity of hnRNP F protein [14]. Here we identified acetylated peptides of two additional proteins hnRNP C1/C2 and hnRNP L, and showed that the acetyl group can be removed by SIRT1 both *in-vitro* and *in-vivo*. Their importance to post-transcription regulation, a basic function that can be involved in multiple biological processes, agrees with the role of SIRT1 in diverse cellular functions. Additionally, hnRNP C1/C2 protein is known to regulate mRNA translation of X-chromosome linked inhibitor of apoptosis (XIAP) to reduce apoptosis [35]. As SIRT1 also mediates apoptosis through p53 [36], it will be interesting to test whether there is genetic interaction between p53 and hnRNP C1/C2.

Our next identified target is RNA-binding motif protein RBM10 that interacts with hnRNP proteins and performs mRNA alternative splicing function [37]. Mutations in *RBM10* cause the TARP syndrome with multiple congenital anomalies. Since the protein is widely present and yet appears to act in a tissue-specific manner, we speculate that deacetylation by SIRT1 might act as a modulating switch to change the activity of RBM10. The fifth protein U4/U6.U5 tri-snRNP-associated protein 1 (SART1) is a component of the spliceosome that performs mRNA splicing. In the liver, SART1 is upregulated during Hepatitis C viral infection in response to interferon-α to promote mRNA expression or alternative splicing of interferon-stimulating genes [38]. We hypothesize that SIRT1 may contribute to the defense mechanism by modifying the acetylation and activity of SART1 during Hepatitis C infection. This provides an additional layer of regulation of important spliceosome proteins of which the activity could fine-tune the magnitude of the anti-viral immune response.

Chromatin-remodeling proteins have already been known to be lysine-acetylated, examples of which are RbAp46 of Sin3a and NuRD deacetylase complexes, and ING4 of a complex containing acetyltransferase HBO1 and PHD finger protein 15 [17]. The identification of SWI/SNF complex subunit SMARCC2 (BAF170) as a specific SIRT1 substrate adds to the broader picture of the extensive role of acetylation in nucleosome disruption and sliding. This result has implications in the study of epigenetics in development and differentiation.

Because the specific lysine residues of the five new SIRT1 substrates have been reported reliably in the mass spectrometry analyses and verified by in-vitro assays, this opens the possibility for the creation of genetic mutants with the corresponding mutations. These site-specific mutations may be less deleterious than null mutations and this allows the careful characterization of phenotype that may otherwise require more complex combinations of tissue-specific Cre and Flox transgenes. Moreover, our identified SIRT1 substrates are RNA-processing proteins that are probably involved in multiple pathways and may give a complex pleiotropic phenotype if the entire gene is removed. It may be useful to create a very specific mutation at the site of the lysine residue to allow the study of the mechanism of acetyl lysine signaling.

Insights into the regulation of SIRT1 has strong medical relevance because disruption of SIRT1 can cause cancer, diabetes and neurodegenerative diseases such as Alzheimer's, Parkinson’s, and Huntington’s diseases [6, 39, 40]. Understanding the lysine acetylome can help in the search for small-molecule SIRT1 modulators that may prevent, slow down, or reverse the progression of these diseases.

## Supporting Information

S1 Fig Validation of SIRT1 target peptides using *in-vitro* deacetylation assay. Peptides examined in this assay were: (1) Acetylated H3 peptide (positive control), (2) Carnitine O-palmitoyltransferase 2 (CPT2) K93ac, (3) Hydroxyacyl-coenzyme A dehydrogenase (HADH) K202ac. Fluorescence level measured the amount of deacetylation. Graphs in this figure are representative of three independent experiments (mean ± SEM) (***P < 0.001).
(EPS)

S1 Table List of the acetylated proteins and peptides identified in *Sirt1* ^*+/+*^ and *Sirt1* ^*-/-*^ mouse livers. (XLSX)

S2 Table List of the acetylation sites tested in the SIRT1 *in vitro* deacetylation assay (mitochondrial and cytosolic proteins). (DOCX)

## References

[1] Deribe YL, Pawson T, Dikic I. Post-translational modifications in signal integration. Nat Struct Mol Biol. 2010;17(6):666–72. Epub 2010/05/25. doi: nsmb.1842 [pii] 10.1038/nsmb.1842 . 20495563

[2] Choudhary C, Weinert BT, Nishida Y, Verdin E, Mann M. The growing landscape of lysine acetylation links metabolism and cell signalling. Nat Rev Mol Cell Biol. 2014;15(8):536–50. Epub 2014/07/24. doi: nrm3841 [pii] 10.1038/nrm3841 . 25053359

[3] Zhao S, Xu W, Jiang W, Yu W, Lin Y, Zhang T, et al Regulation of cellular metabolism by protein lysine acetylation. Science. 2010;327(5968):1000–4. Epub 2010/02/20. doi: 327/5968/1000 [pii] 10.1126/science.1179689 . 20167786 PMC3232675

[4] Berndsen CE, Denu JM. Catalysis and substrate selection by histone/protein lysine acetyltransferases. Curr Opin Struct Biol. 2008;18(6):682–9. Epub 2008/12/06. doi: S0959-440X(08)00156-5 [pii] 10.1016/j.sbi.2008.11.004 . 19056256 PMC2723715

[5] Houtkooper RH, Pirinen E, Auwerx J. Sirtuins as regulators of metabolism and healthspan. Nat Rev Mol Cell Biol. 2012;13(4):225–38. Epub 2012/03/08. doi: nrm3293 [pii] 10.1038/nrm3293 . 22395773 PMC4872805

[6] Bosch-Presegue L, Vaquero A. The dual role of sirtuins in cancer. Genes Cancer. 2011;2(6):648–62. Epub 2011/09/24. 10.1177/1947601911417862 10.1177_1947601911417862 [pii]. . 21941620 PMC3174263

[7] Kelly G. A review of the sirtuin system, its clinical implications, and the potential role of dietary activators like resveratrol: part 1. Altern Med Rev. 2010;15(3):245–63. Epub 2010/12/16. . 21155626

[8] Houtkooper RH, Canto C, Wanders RJ, Auwerx J. The secret life of NAD+: an old metabolite controlling new metabolic signaling pathways. Endocr Rev. 2010;31(2):194–223. Epub 2009/12/17. doi: er.2009-0026 [pii] 10.1210/er.2009-0026 . 20007326 PMC2852209

[9] Purushotham A, Schug TT, Xu Q, Surapureddi S, Guo X, Li X. Hepatocyte-specific deletion of SIRT1 alters fatty acid metabolism and results in hepatic steatosis and inflammation. Cell Metab. 2009;9(4):327–38. Epub 2009/04/10. 10.1016/j.cmet.2009.02.006 19356714 PMC2668535

[10] Brunet A, Sweeney LB, Sturgill JF, Chua KF, Greer PL, Lin Y, et al Stress-dependent regulation of FOXO transcription factors by the SIRT1 deacetylase. Science. 2004;303(5666):2011–5. Epub 2004/02/21. 10.1126/science.1094637 1094637 [pii]. . 14976264

[11] Ponugoti B, Kim DH, Xiao Z, Smith Z, Miao J, Zang M, et al SIRT1 deacetylates and inhibits SREBP-1C activity in regulation of hepatic lipid metabolism. J Biol Chem. 2010;285(44):33959–70. Epub 2010/09/08. doi: M110.122978 [pii] 10.1074/jbc.M110.122978 . 20817729 PMC2962496

[12] Rodgers JT, Lerin C, Haas W, Gygi SP, Spiegelman BM, Puigserver P. Nutrient control of glucose homeostasis through a complex of PGC-1alpha and SIRT1. Nature. 2005;434(7029):113–8. Epub 2005/03/04. doi: nature03354 [pii] 10.1038/nature03354 . 15744310

[13] Sun C, Zhang F, Ge X, Yan T, Chen X, Shi X, et al SIRT1 improves insulin sensitivity under insulin-resistant conditions by repressing PTP1B. Cell Metab. 2007;6(4):307–19. Epub 2007/10/03. doi: S1550-4131(07)00259-8 [pii] 10.1016/j.cmet.2007.08.014 . 17908559

[14] Koumbadinga GA, Mahmood N, Lei L, Kan Y, Cao W, Lobo VG, et al Increased stability of heterogeneous ribonucleoproteins by a deacetylase inhibitor. Biochimica et biophysica acta. 2015. Epub 2015/05/12. doi: S1874-9399(15)00091-7 [pii] 10.1016/j.bbagrm.2015.05.001 . 25959059

[15] Peng L, Ling H, Yuan Z, Fang B, Bloom G, Fukasawa K, et al SIRT1 negatively regulates the activities, functions, and protein levels of hMOF and TIP60. Mol Cell Biol. 2012;32(14):2823–36. Epub 2012/05/16. doi: MCB.00496-12 [pii] 10.1128/MCB.00496-12 . 22586264 PMC3416197

[16] Vaquero A, Scher M, Erdjument-Bromage H, Tempst P, Serrano L, Reinberg D. SIRT1 regulates the histone methyl-transferase SUV39H1 during heterochromatin formation. Nature. 2007;450(7168):440–4. Epub 2007/11/16. doi: nature06268 [pii] 10.1038/nature06268 . 18004385

[17] Kim SC, Sprung R, Chen Y, Xu Y, Ball H, Pei J, et al Substrate and functional diversity of lysine acetylation revealed by a proteomics survey. Mol Cell. 2006;23(4):607–18. Epub 2006/08/19. doi: S1097-2765(06)00454-0 [pii] 10.1016/j.molcel.2006.06.026 . 16916647

[18] Price NL, Gomes AP, Ling AJ, Duarte FV, Martin-Montalvo A, North BJ, et al SIRT1 is required for AMPK activation and the beneficial effects of resveratrol on mitochondrial function. Cell Metab. 2012;15(5):675–90. Epub 2012/05/09. doi: S1550-4131(12)00143-X [pii] 10.1016/j.cmet.2012.04.003 . 22560220 PMC3545644

[19] Yang L, Vaitheesvaran B, Hartil K, Robinson AJ, Hoopmann MR, Eng JK, et al The fasted/fed mouse metabolic acetylome: N6-acetylation differences suggest acetylation coordinates organ-specific fuel switching. Journal of proteome research. 2011;10(9):4134–49. Epub 2011/07/07. 10.1021/pr200313x . 21728379 PMC3204869

[20] Chen Y, Zhao W, Yang JS, Cheng Z, Luo H, Lu Z, et al Quantitative acetylome analysis reveals the roles of SIRT1 in regulating diverse substrates and cellular pathways. Mol Cell Proteomics. 2012;11(10):1048–62. Epub 2012/07/25. doi: M112.019547 [pii] 10.1074/mcp.M112.019547 . 22826441 PMC3494151

[21] Liu TF, Vachharajani V, Millet P, Bharadwaj MS, Molina AJ, McCall CE. Sequential actions of SIRT1-RELB-SIRT3 coordinate nuclear-mitochondrial communication during immunometabolic adaptation to acute inflammation and sepsis. J Biol Chem. 2015;290(1):396–408. Epub 2014/11/19. doi: M114.566349 [pii] 10.1074/jbc.M114.566349 . 25404738 PMC4281742

[22] Trapp J, Meier R, Hongwiset D, Kassack MU, Sippl W, Jung M. Structure-activity studies on suramin analogues as inhibitors of NAD+-dependent histone deacetylases (sirtuins). ChemMedChem. 2007;2(10):1419–31. Epub 2007/07/14. 10.1002/cmdc.200700003 . 17628866

[23] Moynihan KA, Grimm AA, Plueger MM, Bernal-Mizrachi E, Ford E, Cras-Meneur C, et al Increased dosage of mammalian Sir2 in pancreatic beta cells enhances glucose-stimulated insulin secretion in mice. Cell Metab. 2005;2(2):105–17. Epub 2005/08/16. doi: S1550-4131(05)00176-2 [pii] 10.1016/j.cmet.2005.07.001 . 16098828

[24] Tanno M, Sakamoto J, Miura T, Shimamoto K, Horio Y. Nucleocytoplasmic shuttling of the NAD+-dependent histone deacetylase SIRT1. J Biol Chem. 2007;282(9):6823–32. Epub 2007/01/02. 10.1074/jbc.M609554200 . 17197703

[25] Hallows WC, Lee S, Denu JM. Sirtuins deacetylate and activate mammalian acetyl-CoA synthetases. Proc Natl Acad Sci U S A. 2006;103(27):10230–5. Epub 2006/06/23. 10.1073/pnas.0604392103 16790548 PMC1480596

[26] Aquilano K, Vigilanza P, Baldelli S, Pagliei B, Rotilio G, Ciriolo MR. Peroxisome proliferator-activated receptor gamma co-activator 1alpha (PGC-1alpha) and sirtuin 1 (SIRT1) reside in mitochondria: possible direct function in mitochondrial biogenesis. J Biol Chem. 2010;285(28):21590–9. Epub 2010/05/08. 10.1074/jbc.M109.070169 20448046 PMC2898414

[27] Thompson JW, Dave KR, Saul I, Narayanan SV, Perez-Pinzon MA. Epsilon PKC increases brain mitochondrial SIRT1 protein levels via heat shock protein 90 following ischemic preconditioning in rats. PloS one. 2013;8(9):e75753 Epub 2013/09/24. 10.1371/journal.pone.0075753 24058702 PMC3772907

[28] Liu Y, Dentin R, Chen D, Hedrick S, Ravnskjaer K, Schenk S, et al A fasting inducible switch modulates gluconeogenesis via activator/coactivator exchange. Nature. 2008;456(7219):269–73. Epub 2008/10/14. doi: nature07349 [pii] 10.1038/nature07349 . 18849969 PMC2597669

[29] Rodgers JT, Puigserver P. Fasting-dependent glucose and lipid metabolic response through hepatic sirtuin 1. Proc Natl Acad Sci U S A. 2007;104(31):12861–6. Epub 2007/07/25. doi: 0702509104 [pii] 10.1073/pnas.0702509104 . 17646659 PMC1937557

[30] Dominy JE Jr., Lee Y, Gerhart-Hines Z, Puigserver P. Nutrient-dependent regulation of PGC-1alpha's acetylation state and metabolic function through the enzymatic activities of Sirt1/GCN5. Biochimica et biophysica acta. 2010;1804(8):1676–83. Epub 2009/12/17. 10.1016/j.bbapap.2009.11.023 20005308 PMC2886158

[31] Frescas D, Valenti L, Accili D. Nuclear trapping of the forkhead transcription factor FoxO1 via Sirt-dependent deacetylation promotes expression of glucogenetic genes. J Biol Chem. 2005;280(21):20589–95. Epub 2005/03/25. 10.1074/jbc.M412357200 . 15788402

[32] Walker AK, Yang F, Jiang K, Ji JY, Watts JL, Purushotham A, et al Conserved role of SIRT1 orthologs in fasting-dependent inhibition of the lipid/cholesterol regulator SREBP. Genes Dev. 2010;24(13):1403–17. Epub 2010/07/03. 10.1101/gad.1901210 20595232 PMC2895199

[33] Carpenter B, MacKay C, Alnabulsi A, MacKay M, Telfer C, Melvin WT, et al The roles of heterogeneous nuclear ribonucleoproteins in tumour development and progression. Biochimica et biophysica acta. 2006;1765(2):85–100. Epub 2005/12/28. doi: S0304-419X(05)00063-6 [pii] 10.1016/j.bbcan.2005.10.002 . 16378690

[34] Chaudhury A, Chander P, Howe PH. Heterogeneous nuclear ribonucleoproteins (hnRNPs) in cellular processes: Focus on hnRNP E1's multifunctional regulatory roles. RNA. 2010;16(8):1449–62. Epub 2010/06/30. doi: rna.2254110 [pii] 10.1261/rna.2254110 . 20584894 PMC2905745

[35] Holcik M, Gordon BW, Korneluk RG. The internal ribosome entry site-mediated translation of antiapoptotic protein XIAP is modulated by the heterogeneous nuclear ribonucleoproteins C1 and C2. Mol Cell Biol. 2003;23(1):280–8. Epub 2002/12/17. . 12482981 10.1128/MCB.23.1.280-288.2003 PMC140676

[36] Vaziri H, Dessain SK, Ng Eaton E, Imai SI, Frye RA, Pandita TK, et al hSIR2(SIRT1) functions as an NAD-dependent p53 deacetylase. Cell. 2001;107(2):149–59. Epub 2001/10/24. doi: S0092-8674(01)00527-X [pii]. . 11672523 10.1016/s0092-8674(01)00527-x

[37] Wang Y, Gogol-Doring A, Hu H, Frohler S, Ma Y, Jens M, et al Integrative analysis revealed the molecular mechanism underlying RBM10-mediated splicing regulation. EMBO Mol Med. 2013;5(9):1431–42. Epub 2013/09/04. 10.1002/emmm.201302663 . 24000153 PMC3799496

[38] Lin W, Zhu C, Hong J, Zhao L, Jilg N, Fusco DN, et al The spliceosome factor SART1 exerts its anti-HCV action through mRNA splicing. J Hepatol. 2015;62(5):1024–32. Epub 2014/12/08. doi: S0168-8278(14)00887-3 [pii] 10.1016/j.jhep.2014.11.038 . 25481564 PMC4404186

[39] Herskovits AZ, Guarente L. Sirtuin deacetylases in neurodegenerative diseases of aging. Cell Res. 2013;23(6):746–58. Epub 2013/05/22. doi: cr201370 [pii] 10.1038/cr.2013.70 . 23689277 PMC3674397

[40] Huynh FK, Hershberger KA, Hirschey MD. Targeting sirtuins for the treatment of diabetes. Diabetes Manag (Lond). 2013;3(3):245–57. Epub 2013/05/01. 10.2217/dmt.13.6 . 25067957 PMC4110209

