# Supplementary material for: Acetylome Analysis Identifies SIRT1 Targets in mRNA-Processing and Chromatin-Remodeling in Mouse Liver
Source: PLoS One. 2015 Oct 15;10(10):e0140619. doi: 10.1371/journal.pone.0140619 (PMC4607365; doi:10.1371/journal.pone.0140619)
Supplement: S2 Table — (DOCX) [file pone.0140619.s003.docx]

**S2 Table. List of the acetylation sites tested in the SIRT1 *in vitro* deacetylation assay (mitochondrial and cytosolic proteins).**

| **Accession #** | **Uniprot #** | **Protein name** | **Peptide sequence** | **Acetylation site** | **Cellular component** | **Biological process** |
| --- | --- | --- | --- | --- | --- | --- |
| IPI00881401.1 | P52825 | carnitine O-palmitoyltransferase 2(CPT2) | ENGIG(**AcK**)ELHAH | K93 | Mitochondrion,  Nucleus | Fatty acid metabolism |
| IPI00121105.2 | Q61425 | Hydroxyacyl-coenzyme A dehydrogenase (HADH) | LVDFC(**AcK**)TLGKH | K202 | Mitochondrion, Nucleus | Fatty acid metabolism |
| IPI00471246.2 | Q9JHI5 | Isovaleryl-CoA dehydrogenase (IVD) | QTNDF(**AcK**)NLREF | K76 | Mitochondrion | Fatty acid metabolism |
| IPI00323592.2 | P08249 | Malate dehydrogenase (MDH2) | NLGIG(**AcK**)ITPFE | K307 | Mitochondrion,  Nucleus | TCA cycle |
| IPI00468481.2 | P56480 | ATP synthase subunit beta (ATP5b) | GVINL(**AcK**)DATSK | K259 | Mitochondrion  Nucleus | ATP synthesis,  Ion transport, |
| IPI00468481.2 | O08756 | 3-hydroxyacyl-CoA dehydrogenase type-2 (HSD17B10) | TTLPE(**AcK**)VRNFL | K212 | Mitochondrion, Plasma membrane,  ER | Fatty acid metabolism |
| IPI00119114.2 | P51174 | Long-chain specific acyl-CoA dehydrogenase (LCAD) | RKAFG(**AcK**)TVAHI | K322 | Mitochondrion,  Cytosol | Fatty acid metabolism, |
